# Atypical Presentation of a Hemorrhagic Glioblastoma Multiforme Mimicking a Cerebral Contusion

**DOI:** 10.7759/cureus.39592

**Published:** 2023-05-28

**Authors:** Guilherme S Piedade, Eni Manoku, Andreas L Gelhardt, Joacir G Cordeiro, Jorge A Terzis

**Affiliations:** 1 Department of Neurosurgery, Helios Universitätsklinikum Wuppertal, Wuppertal, DEU; 2 Department of Neurosurgery, Medical School, Federal University of Paraná, Curitiba, BRA; 3 Department of Neurological Surgery, University of Miami, Coral Gables, USA

**Keywords:** hemorrhage, traumatic brain injury, mri, cerebral contusion, glioblastoma multiforme

## Abstract

The emergency room management of a patient with external signs of cranial trauma and imaging showing brain hemorrhage can be dangerously misleading. This case of a patient with glioblastoma could only be timely diagnosed because of cautious evaluation of imaging findings. A 60-year-old patient presented to the emergency room after being found down with external signs of cranial trauma and a reduced level of consciousness. Computed tomography revealed a right frontal polar cortical hemorrhage of around 12 mm diameter with no perilesional edema or contrast enhancement. Likewise, the MRI showed no contrast enhancement. Before the scheduled MRI follow-up was performed the patient became symptomatic leading to an earlier repeat that showed massive progression. She underwent surgical resection that revealed the lesion to be an aggressive glioblastoma. High suspicion of an underlying neoplastic lesion in atypical brain hemorrhage in trauma patients is paramount. Short MRI follow-up is recommended as soon as the hematoma resorbs to prevent delays with potential impact or patient outcome.

## Introduction

Glioblastoma multiforme typically presents with seizures, headache or focal neurological deficits [1]. Symptoms trigger diagnostic neuroimaging, revealing a brain tumor with heterogeneous patterns of contrast enhancement [1]. The diagnosis of a brain tumor in the setting of cranial trauma and altered mental state may be cumbersome. The presence of hemorrhage on a frontal polar area with no other associated findings may be misleading and rise the suspicion of traumatic origin. This could lead to a dangerous delay in the histological diagnosis and treatment with potential impact on neurologic outcome. This case of a patient with atypical imaging presentation of a glioblastoma discusses the management of imaging findings in the setting of acute cranial trauma.

## Case presentation

**Figure 1:**
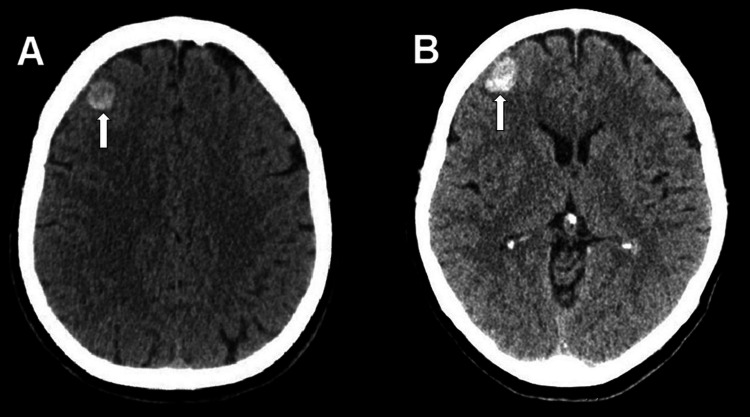
Axial CT images at admission in the emergency room (A) and six hours later (B) showing a round hemorrhagic lesion

A 60-year-old female presented to the emergency room after being found down. Emergency medical service reported a brief generalized tonic-clonic seizure en route. Upon ER arrival, her Glasgow coma score was 9 (E3+ V1+ M5) and the neurologic exam had no localization signs. Supraorbital laceration and periorbital hematoma were observed. Brain CT revealed a frontal polar cortical hemorrhage of around 12 mm diameter with no perilesional edema (Figure 1). The contrast-enhanced phase showed no contrast enhancement. The six hours repeat CT showed mild hemorrhage progression. In the same admission, the patient was again neurologically intact, but with retrograde amnesia regarding the trauma.

**Figure 2:**
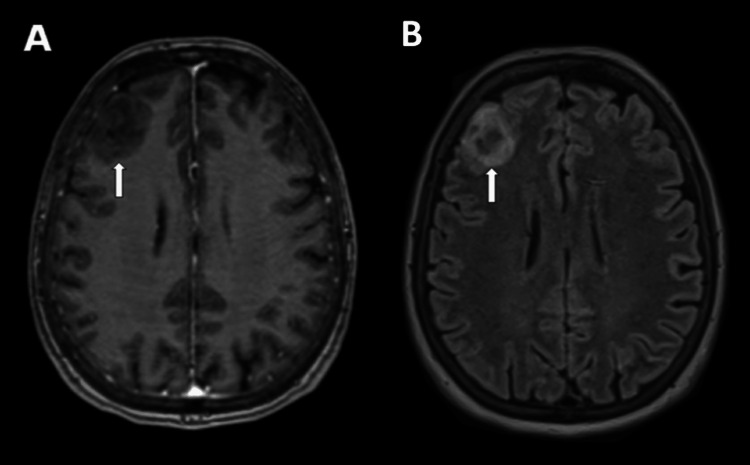
Axial MRI images on the day following admission showing no contrast enhancement (A), but perifocal edema in FLAIR (B) FLAIR: fluid-attenuated inversion recovery

The hypothesis of a cortical venous thrombosis was brought by the contrast-enhanced CT scan, hence a brain MR venography was performed. The thrombosis was not confirmed and the frontal lesion showed to be hyperintense in T2 and hypointense in T1 with mild perilesional edema, with abnormal susceptibility and no diffusion restriction or contrast enhancement (Figure 2). The patient was discharged home with a repeat CT scheduled in two weeks and a follow-up MRI with contrast was planned to be done in around three months.

**Figure 3:**
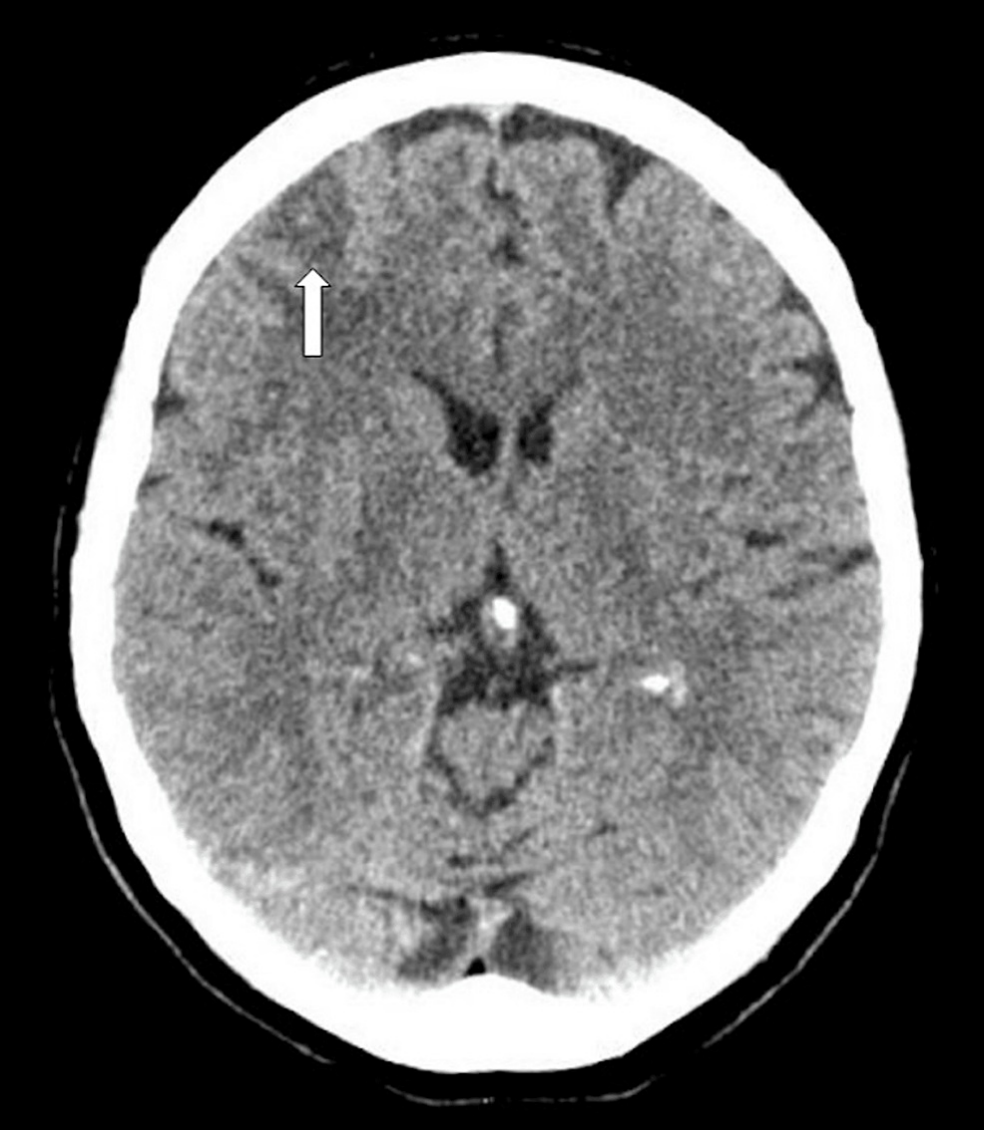
Axial CT image two weeks following the first presentation documented resorption of the blood and a progressive perifocal edema

**Figure 4:**
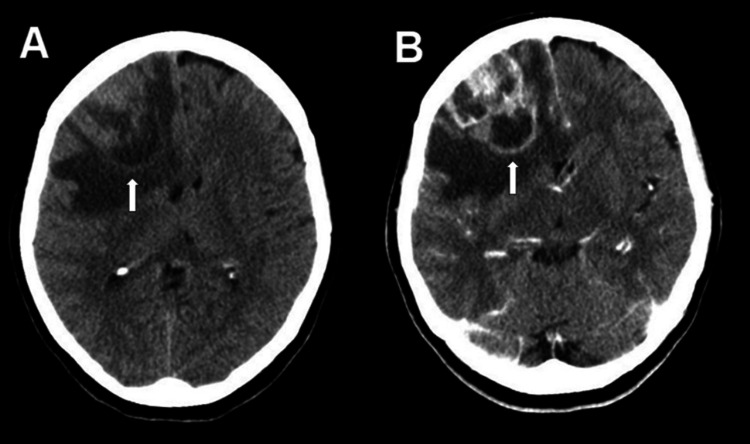
Axial CT performed because of headache revealed a large right frontal mass with perifocal edema (A) and contrast enhancement (B)

**Figure 5:**
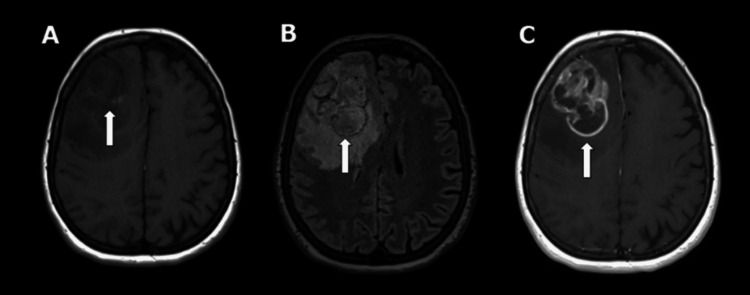
Axial MRI images showing a large right frontal mass in T1 (A) with perifocal edema in FLAIR (B) and contrast enhancement (C) FLAIR: fluid-attenuated inversion recovery

Two weeks later, a CT scan showed reabsorption of the blood with persistent perilesional edema (Figure 3). After two months and three weeks, shortly before the scheduled MRI, the patient visited a neurologist and complained of headaches. A CT scan revealed a large right frontal mass with massive perifocal edema. The scan was complemented with a contrast phase and enhancement was seen (Figure 4). The MRI this time confirmed a large lesion with contrast enhancement, progressive edema, and midline shift (Figure 5). The patient was admitted on the same day for tumor resection. The histological examination revealed glioblastoma with IDH-wild type and the unmethylated MGMT promoter. She underwent concomitant radiation and chemotherapy with temozolomide following the Stupp protocol. In the last month of chemotherapy, she presented another seizure and hemiplegia. Repeat MRI showed tumor progression that led to a second resection six months after the first procedure. The patient was later lost to follow-up.

## Discussion

Intratumoral hemorrhage has an incidence of 6.5-8% in glioblastoma, much lower than in metastatic brain tumors [2,3]. When it happens in a setting of suspected traumatic brain injury, it may mimic a cerebral contusion, like in this case. Depending on tumor location, a hemorrhagic glioblastoma may even mimic a hypertensive intracerebral hemorrhage [4,5]. Contrast studies may not reveal enhancement in the early phase of the bleeding; enhancement may also not be present if the hemorrhagic tumor has still a lower grade at the time of presentation [6].

Some features of this case allowed a correct and timely diagnosis in spite of the bleeding as a confounding factor. The most important was the perilesional edema, evidenced in the first MRI and progressive in the ensuing imaging studies. Considering the anamnesis with possible trauma, a CT scan was certainly the indicated imaging modality at presentation, but the possible postictal status with a later short seizure in the presence of the paramedics would later require an MRI either way.

The finding in the initial CT scan, when interpreted as cerebral contusion, had no sure surgical indication. However, in the case that a resection had been performed, a histopathological examination of the hematoma may have detected the glioblastoma. This reinforces the need for neuropathological examination of every specimen resected from the brain, specially hematomas, even when a hypertensive intracerebral hemorrhage in a typical location is the most probable diagnosis.

The diagnosis of a brain tumor in this case was not possible at the presentation when following a reasonable decision-making process. The early MRI showing no contrast enhancement lowered the possibility of a brain tumor at that moment and the recommendation of a follow-up after resolution of the bleeding was adequate. MRI is important in the investigation of an atypical hemorrhage, but it does not always indicate its cause, as in this case. As an investigation tool, it should be primarily indicated when the cause of the bleeding is not clear.

This patient suffered from a de novo IDH-wild-type glioblastoma, which is the most aggressive form of this tumor [7]. The unfavorable molecular classification explains the massive progress seen when both MRIs are compared. Seizures may lead to hemorrhagic transformation in this tumor and mask the real diagnosis in imaging. In such cases, short follow-ups are essential. Considering the confounding factors, the diagnosis was not significantly delayed in our case and the patient underwent state-of-the-art therapy in a timely manner.

## Conclusions

This case highlights the challenges in diagnosing a brain tumor in the setting of suspected traumatic brain injury. Hemorrhagic transformation may mask a plethora of important differential diagnoses for intracerebral hemorrhages. The presence of progressive perilesional edema in this case raised suspicion for an underlying pathology. If contrast studies are normal, selected patients may benefit from shorter follow-up imaging after resolution of the bleeding in selected cases.
